# When gender matters: inequalities in health services utilization and risk factors monitoring after acute myocardial infarction

**DOI:** 10.3389/fgwh.2025.1605400

**Published:** 2025-06-26

**Authors:** Irene López-Ferreruela, Antonio Gimeno-Miguel, Clara Laguna-Berna, Sara Malo, Sara Castel-Feced, María José Rabanaque, Isabel Aguilar-Palacio

**Affiliations:** ^1^ Torreramona Health Centre, Primary Care, Servicio Aragonés de Salud (SALUD), Zaragoza, Spain; ^2^ Grupo de Investigación en Servicios Sanitarios de Aragón (GRISSA), Fundación Instituto de Investigación Sanitaria de Aragón (IIS Aragón), Zaragoza, Spain; ^3^ EpiChron Research Group, Aragon Health Sciences Institute (IACS), IIS Aragón, Miguel Servet University Hospital, Zaragoza, Spain; ^4^ Research Network on Chronicity, Primary Care and Health Promotion (RICAPPS), Carlos III Health Institute (ISCIII), Madrid, Spain; ^5^ Department of Preventive Medicine and Public Health, University of Zaragoza, Zaragoza, Spain; ^6^ Department of Statistical Methods, University of Zaragoza, Zaragoza, Spain

**Keywords:** gender inequalities, delivery of healthcare, healthcare utilization, cardiovascular risk factors, myocardial infarction, secondary prevention, real-world data

## Abstract

**Introduction:**

Secondary prevention after an acute myocardial infarction (AMI) has the objective of improving quality of life, minimizing recurrence, and reducing morbidity and mortality. Despite European guidelines highlighting the importance of cardiovascular risk factor (CVRF) management and optimal healthcare utilization, inequalities persist, particularly between genders. This study aims to identify and analyze gender inequalities in healthcare utilization and CVRF monitoring during the first year after AMI using real-world data (RWD).

**Methods:**

An analytical study was conducted within the CARhES (CArdiovascular Risk factors for Health Services research) cohort in Aragon, Spain. The study population included 3,464 subjects who survived a first AMI and were followed for one full year after the event. Sociodemographic, anthropometric, clinical data, healthcare utilization, CVRF monitoring and pharmacological prescriptions, were extracted from the Aragon Health Service. Statistical analyses included chi-squared tests, Student's *t*-tests, and logistic regression, with Blinder-Oaxaca decomposition applied to explore possible explanatory factors for gender differences.

**Results:**

Women represented 28.3% of the study population. Compared with men, they were older and had a higher morbidity burden. Primary care utilization was similar between genders; however, women had fewer cardiology visits (*p* < 0.001) and were less likely to achieve risk factor monitoring goals. Differences were also observed in pharmacological treatment, with women being less likely to receive beta-blockers, lipid modifying agents, and antiplatelet agents (*p* < 0.001). Several of these inequalities persisted after controlling for age. The Oaxaca decomposition showed that age and morbidity burden were the main contributors to gender disparities. In addition, socioeconomic status and place of residence played a role in health services utilization differences.

**Conclusions:**

Gender inequalities are still present in post-AMI care and CVRF management, with women being more likely to receive less adequate treatment and management. Addressing these inequalities is crucial to ensuring equitable care and improving health outcomes for women.

## 1 Introduction

The primary objective of secondary prevention following an acute myocardial infarction (AMI) is to enhance patient's quality of life and to minimize recurrence, reduce morbidity and mortality rates (1–3). In order to obtain these outcomes, European guidelines emphasise the importance of following healthy lifestyle recommendations, effective management and monitoring of cardiovascular risk factors (CVRF), such as blood pressure, cholesterol, and glucose levels, and the appropriate use of recommended drugs, such as platelet aggregation inhibitors, beta-blockers, lipid modifying agents, renin–angiotensin–aldosterone system inhibitors and other comedications (4–6). Patients must be followed up properly, particularly during the first year after an AMI, in order to ensure the optimal utilization of healthcare services and the establishment of a positive, collaborative, and trusting therapeutic relationship that supports patient adherence, recovery, and commitment to their health (7).

Despite significant efforts to highlight the importance of CVRF management, a concerning number of patients fail to achieve the recommended targets (8).

In this context, recent evidence has suggested the existence of gender inequalities, defined as systematic, avoidable, and unjust differences in health status and healthcare access based on gender (9). Gender norms, roles, and power imbalances have been demonstrated to shape vulnerabilities to illness, influence health behaviours and care-seeking, and affect access to health services, treatment responses, and health outcomes (9).

As supported by research in cardiovascular disease, women are less likely than men to receive adequate risk factors assessment during secondary prevention and show poorer monitoring and achievement of key risk factors targets (such as glycated haemoglobin and lipoprotein cholesterol). They are also less likely to achieve guideline recommendations, including sufficient levels of physical activity, or being more frequently obese (1,8).

Moreover, gender inequalities have been documented even before healthcare contact. Qualitative studies have shown that women are more likely to misinterpret or minimize their symptoms, delay seeking medical attention, and experience greater uncertainty during the decision-making process, often attributing symptoms to non-cardiac causes and contributing to worse outcomes. Conversely, men have been shown to recognize the urgency of their symptoms and seek care earlier than women (10–12). These inequalities highlight gaps not only in preventive care, but throughout the entire care pathway.

Despite increasing awareness, research in this area remains limited. Additionally, the growing availability of real-world data (RWD), defined as routinely collected health information from sources such as electronic health records, administrative databases, or patient registries, presents a unique opportunity to enhance clinical research (13). When appropriately analysed, RWD can generate real-world evidence (RWE), providing valuable insights into healthcare utilization, treatment patterns, and outcomes in everyday clinical settings. This enables performing large-scale population secondary studies that can offer valuable evidence on gender inequalities in healthcare. Therefore, the objective of this study is to compare, describe and analyse, using RWD, the level of health services utilization and the monitoring of CVRFs between men and women during the first year after an AMI, in order to identify potential gender inequalities and explore their underlying causes.

## 2 Materials and methods

### 2.1 Study design

An analytical study based on observational population data was conducted within the CARhES (CArdiovascular Risk factors for hEalth Services research) cohort. This is an open, dynamic, population-based cohort of subjects aged 16 and older diagnosed with hypertension, diabetes mellitus, and/or dyslipidaemia in the Spanish region of Aragon (14).

### 2.2 Study population

**Figure 1:**
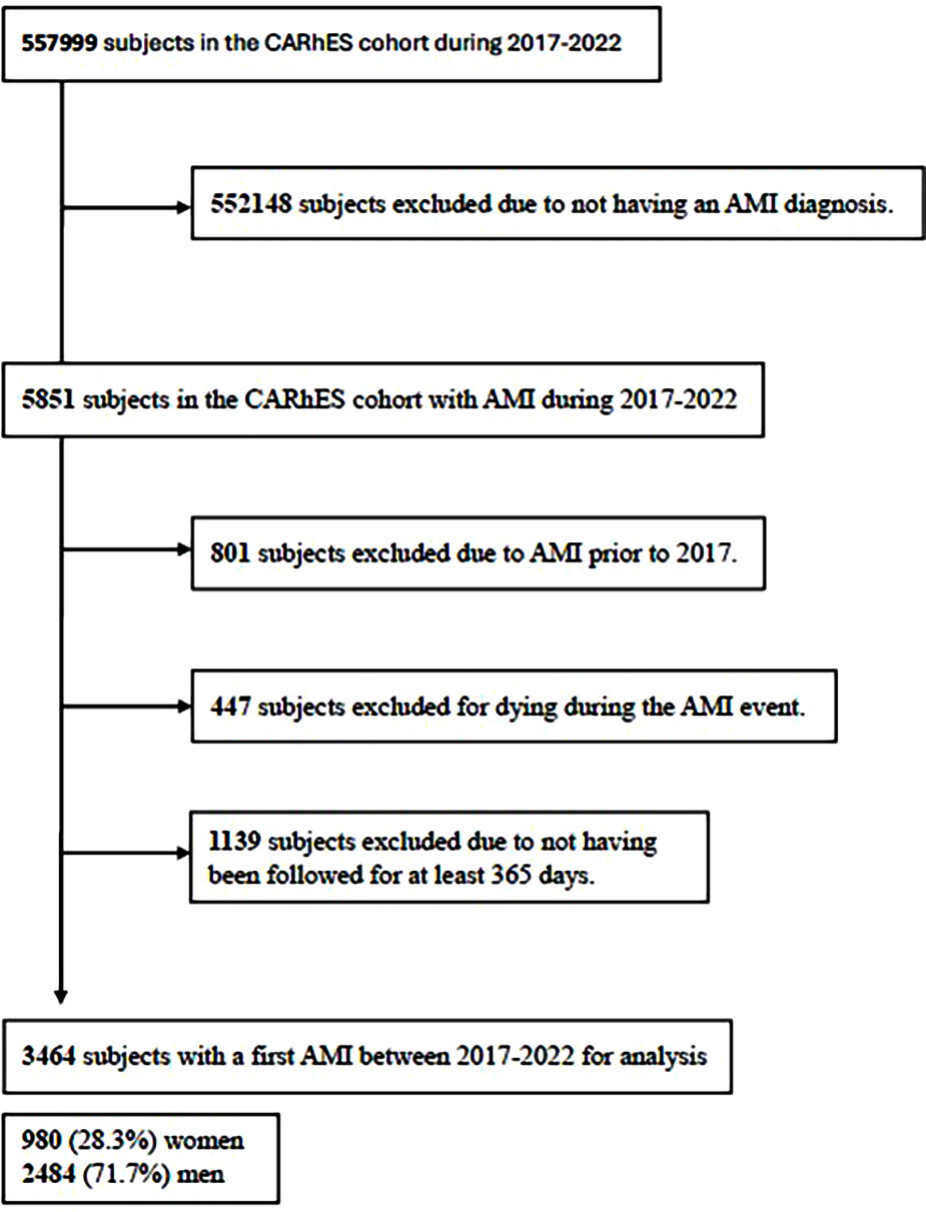
Study flowchart of subjects’ inclusion and exclusion for the study. CARhES, CArdiovascular Risk factors for hEalth Services research. AMI, acute myocardial infarction.

For the purposes of this study, we included patients from the CARhES cohort who experienced a first recorded episode of AMI between 2017 and 2022 identified using the International Classification of Diseases (ICD-10) code I21. We excluded subjects with a prior diagnosis of AMI at the onset of cohort follow-up, as well as those who died during the index event. In order to analyse health services utilization and CVRF monitoring, we only included subjects with complete clinical and administrative data available for the 365 days following the AMI event. A detailed flowchart illustrating selection criteria of the study population is provided in Figure 1.

### 2.3 Data sources

The data used in this study were obtained from the CARhES cohort (14), a population-based, dynamic open cohort designed to analyse the impact of healthcare service use and pharmacological treatment on health outcomes in patients with CVRFs. The CARhES cohort is constructed using RWD, that is, routinely collected health information from clinical practice, including all individuals aged ≥16 years registered in the Aragón public health system (Spain) with a diagnosis of hypertension, diabetes mellitus, and/or dyslipidaemia since 2017. The cohort is subject to annual updates through data extractions.

This study constitutes a secondary analysis of anonymized RWD extracted from the BIGAN platform, which integrates multiple information systems of the Aragón Health Service (SALUD) for research and policy purposes. The integrated sources include the following: the user database (BDU) and adjusted morbidity groups (GMA) for demographic and clinical data; hospital discharge records (CMBD); specialist care data (CEX); primary care records (OMI-AP); emergency care data (PCH); and electronic pharmacy dispensing records (Receta Electrónica) for medication use. Collectively, these sources provide comprehensive information on patients' clinical profiles, socioeconomic conditions, and healthcare utilization. No additional instruments or patient-reported outcome measures were used; all data were obtained from routinely recorded clinical and administrative sources within the public health system.

### 2.4 Variables

In this study, the variables were grouped into two main categories: general patient characteristics at the time of the event (such as socio-demographic, anthropometric and clinical characteristics) and patient management variables, which included health service utilization, CVRF monitoring, and pharmacological treatment during the first year following AMI. The sex/gender variable was also included, as a key factor for analysing potential gender-based differences in care and outcomes.

#### 2.4.1 General patient characteristics

##### 2.4.1.1 Sociodemographic variables

Sociodemographic and anthropometric data were recorded at the time of the event. This included age, gender, nationality (classified as Spanish or immigrant), area of residence (urban or rural, according to the basic healthcare area in which the subject resided), institutionalisation in a nursing home, and socioeconomic status. Socioeconomic status was defined according to the income category of the subject. This included pensioners with income below 18,000€ per year and free pharmacy, pensioners with income above 18,000€ per year, unemployed, subjects with active employment with income below 18,000€ per year, active with income above 18,000€ per year, and other status (including mutual, special conditions or uninsured subjects).

##### 2.4.1.2 Anthropometric variables

Weight (kg) and height (cm) were recorded to calculate the body mass index (BMI), defined as weight in kilograms divided by the height in metres squared, and then categorized in accordance with the World Health Organization (WHO) classification (15). A BMI lower than 18.5 kg/m^2^ was defined as underweight, while a normal range was established between 18.5 kg/m^2^ and 24.9 kg/m^2^. A BMI between 25 kg/m^2^ and 29.9 kg/m^2^ was defined as overweight, and a BMI of 30 kg/m^2^ or above was defined as obese.

##### 2.4.1.3 Clinical characteristics

In terms of clinical information, we obtained several variables from the Morbidity adjusted groups (GMA). This is an information source that includes all medical diagnoses available in primary care, emergencies and hospital discharge records (Minimum Basic Data Set of Hospital Discharges) (16). It provides a description of the main comorbidities of each subject, a numeral quantification of their chronic pathologies, the subject's complexity, which is defined by the analysis of several resource utilization variables, such as mortality, risk of hospitalization, primary care visits, or prescriptions, linked to diagnoses, and their morbidity burden, obtained from the aggregation of the patient's different diagnoses.

Additionally, CVRF that were required for inclusion in the CARhES cohort (hypertension, diabetes mellitus and dyslipidemia) were registered.

#### 2.4.2 Patient management variables

##### 2.4.2.1 Health services utilization

In order to measure health services utilization among our population, the number of visits recorded in the database was quantified. From this basis, it was calculated the proportion of subjects who had visited at least once the primary care services, including general practitioner and nurse and specialist healthcare. In specialist healthcare, we specifically evaluated visits to cardiologist, endocrinologist, vascular surgeon, nephrologist and ophthalmologist, hospital admission and the emergency room use in the year following AMI.

##### 2.4.2.2 CVRF monitoring

*CVRF* monitoring was assessed by monitoring the proportion of subjects who had at least one measurement for each of the following measures: blood pressure, capillary blood glucose, glycosylated haemoglobin (HbA1c), cholesterol values in blood tests, waist circumference, electrocardiogram (ECG), influenza vaccination and diabetic foot risk measured, as well as their self-report on adequate nutrition, physical activity and adherence to treatment.

##### 2.4.2.3 Pharmacological treatment

Pharmacological treatment was selected in accordance with the recommendations set out in the European guidelines (4–6),identified by anatomical therapeutic chemical (ATC)-code, version 2024 (17), and defined based on prescriptions registered during the study year. This data does not reflect actual dispensation or therapy initiation. Pharmacological burden was included, defined as the number of different pharmacological subgroups that the individual was prescribed and dispensed during the study year. So, we included antihypertensive drugs (ATC-code C02), diuretics (C03), beta-blockers (C07) (all beta-blocking agents and combinations), calcium channel blockers (CCBs) (C08), angiotensin-converting enzyme inhibitors (ACE-I)/angiotensin receptor blocker (ARB) (C09), lipid modifying agents (C10), antiplatelets agents (B01AC), vitamin K antagonist (B01AA), direct thrombin inhibitors (B01AE) and direct factor Xa inhibitors (B01AF). The proportion of subjects who received at least one prescription for the selected drugs in the year following AMI was calculated.

#### 2.4.3 Sex and gender

In our study, the variable measured was biological sex, as recorded in the health information systems. However, for analytical and interpretative purposes, the term gender is used, acknowledging that it encompasses a broader set of socio-cultural norms, roles, and behaviours that influence health outcomes. Furthermore, gender intersects with other axes of inequality, including socioeconomic status, place of residence, and access to care.

Although it is not always possible to disentangle the effects of sex and gender, the use of the term gender inequality reflects the intention to consider the structural and behavioural dimensions beyond biology. The assessment of gender inequality was conducted through a comparative analysis of frequency data and a subsequent calculation of adjusted rates. This approach was undertaken under the assumption that men and women should have equal access to healthcare services and similar levels of CVRFs monitoring. Any statistically significant deviation from this expected equality was interpreted as an indication of gender inequality.

### 2.5 Statistical analyses

Sociodemographic and baseline clinical characteristics of the subjects studied were described using counts and proportions for categorical variables and means with standard deviation (SD) for continuous variables. Bivariate analyses between men and women were performed using Pearson's Chi-squared test, and we compared means between groups using Student's *T*-test. Bivariate logistic regression analyses were conducted to investigate gender-based differences in patient management variables that had been previously defined. The threshold for statistical significance was set at *P* < 0.05. When statistically significant gender-based differences were identified, the Blinder-Oaxaca decomposition method was applied (18). A twofold decomposition was conducted using the Oaxaca R library and reference regression coefficients, which were calculated from a pooled regression model (19). Variables such as age, socioeconomic status, area of residence (urban or rural), and morbidity burden were examined to determine their contribution to the observed differences. This analytical approach enables the quantification of the extent to which observed gender differences can be attributed to measurable variables, and how much remains unexplained. Specifically, it decomposes the mean outcome differences between two groups into an explained fraction—linked to differences in observed characteristics—and an unexplained fraction, which may reflect the differential effects of those variables, unmeasured confounders, behavioural or structural inequalities, or potential discrimination (20). This method provides a nuanced understanding of the mechanisms underlying gender disparities in healthcare. All statistical analyses were performed using R 4.3.3. (R Core Team, 2022) a language and environment for statistical computing. R Foundation for Statistical Computing, Vienna, Austria. URL https://www.R-project.org/) (21) and JAMOVI (version 2.4) [Computer Software) Retrieved from https://www.jamovi.org (22).

### 2.6 Ethical aspects

This study is based on data from the CARhES cohort, whose protocol was approved by the Clinical Research Ethics Committee of Aragon (CEICA PI21/148). The research was conducted in accordance with local legislation and institutional requirements. As the study involved the retrospective analysis of anonymized, population-based data with no direct contact or interaction with participants, the requirement for written informed consent was waived by the Ethics Committee.

## 3 Results

### 3.1 General characteristics of the study population

The study population included a total of 3,464 subjects who had experienced a first AMI during 2017–2022, with a minimum follow-up of one year. Of these, 980 (28.30%) were women, and 2,482 (71.70%) were men.

As presented in Table 1, women were significantly older at the time of the event (75.26 years) compared to men (67.23 years). The majority of the population were pensioners <18,000€, with significant differences (*p* < 0.001) observed between men (41.79%) and women (67.45%). 2,471 subjects (71.33%) lived in urban areas. A greater proportion of women (10.92%) were institutionalized in nursing homes compared to men (4.31%).

**Table 1:** General patient characteristics (socio-demographic, clinical and anthropometric). Results overall and stratified by gender.

*N*, %	Overall		Women		Men		*p* values
Population	3,464	100.00	980	28.30	2,484	71.70	<0.001
Age at the event (mean, sd)*	69.49	13.01	75.26	12.42	67.23	12.54	<0.001
Nationality							
Spanish	3,317	95.78	957	97.65	2,360	95.01	<0.001
Immigrant	146	4.22	23	2.35	123	4.95	
Socioeconomic status							
Pensioners <18,000€ per year	1,699	49.05	661	67.45	1,038	41.79	<0.001
Pensioners >18,000€ per year	698	20.15	147	15.00	551	22.18	
Unemployed	173	4.99	42	4.29	131	5.27	
Actives <18,000€ per year	384	11.09	56	5.71	328	13.20	
Actives >18,000€ per year	387	11.17	37	3.78	350	14.09	
Other socioeconomic level	123	3.55	37	3.78	86	3.46	
Residential area							
Urban	2,471	71.33	728	74.29	1,743	70.17	0.016
Rural	993	28.67	252	25.71	741	29.83	
Institutionalized	214	6.18	107	10.92	107	4.31	<0.001
Comorbidities							
Hypertension	2,417	69.77	777	79.29	1,640	66.02	<0.001
Dyslipidemia	3,407	98.35	950	96.94	2,457	98.91	<0.001
Diabetes Mellitus	1,707	49.28	475	48.47	1,232	49.60	0.550
Heart failure	392	11.32	169	17.24	223	8.98	<0.001
Chronic Obstructive							
Pulmonary Disease	328	9.47	61	6.22	267	10.75	<0.001
Depression	542	15.65	251	25.61	291	11.71	<0.001
Chronic Kidney Disease	744	21.48	247	25.20	497	20.01	<0.001
Cirrhosis	112	3.23	30	3.06	82	3.30	0.723
Osteoporosis	271	7.83	245	25.00	26	1.05	<0.001
Dementia	107	3.09	65	6.63	42	1.69	<0.001
N° Pathologies (mean, sd)*	6.43	2.86	7.49	2.90	6.01	2.73	<0.001
Complexity (mean, sd)*							
Level 1 (minimum)	213	6.15	42	4.29	171	6.88	0.002
Level 5 (maximum)	615	17.76	152	15.51	463	18.64	
Morbidity burden (mean, sd)*	12.10	6.11	14.00	6.22	11.35	5.90	<0.001
Body Mass Index (Missing values: 1,103)							
Underweight	14	0.59	7	1.09	7	0.41	<0.001
Normal range	447	18.93	154	23.95	293	17.05	
Overweight	1,063	45.02	247	38.41	816	47.50	
Obese	837	35.45	235	36.55	602	35.04	
Smoking habit	573	20.51	115	4.12	458	16.39	<0.001

*N*, number %: percentage; p, statistical significance *p* < 0.05. Pearson's Chi-squared test. Student's *T*-test.

* Continuous variables expressed as mean, standard deviation (sd).

While dyslipidemia was the most prevalent CVRF overall, with a slightly higher prevalence among men, hypertension was significantly more common in women. Among the comorbidities analyzed, all conditions (except cirrhosis and chronic obstructive pulmonary disease) were more prevalent in women (*p* < 0.001). Women exhibited a significantly higher number of pathologies, more affected systems, and a greater overall morbidity burden compared to men (*p* < 0.001). Conversely, men had higher rates of overweight and obesity and had a higher prevalence of smoking (*p* < 0.001) (Table 1).

### 3.2 Health services utilization

Nearly all participants (99.86%) had at least one primary care visit within the first year after AMI (Table 2). Women were more likely to visit nurse practitioners (93.67% vs. 91.77%) and specialists such as endocrinologists and ophthalmologists (*p* < 0.001),while men had significantly more visits to cardiologists (*p* < 0.001). Hospital admissions by all the causes were significantly higher in women (35.10% vs. 30.76%), while emergency room use was similar between genders (Table 2).

**Table 2:** Patient management: health services utilization, risk factors monitoring and pharmacological treatment.

*N*, %	Overall (*n* = 3,464)		Women (*n* = 980)		Men (*n* = 2,484)		*p* values
People with at least one visit							
Primary care	3,459	99.86	979	99.90	2,480	99.84	0.680
General practitioner	3,454	99.71	976	99.59	2,478	99.76	0.410
Nurse	3,179	91.77	918	93.67	2,261	91.02	0.011
Specialty care	3,421	98.76	963	98.27	2,458	98.95	0.100
Cardiologist	3,298	95.21	911	92.96	2,387	96.10	<0.001
Endocrinologist	362	10.45	116	11.84	246	9.90	0.094
Vascular surgeon	199	5.74	47	4.80	152	6.12	0.132
Nephrologist	200	5.77	54	5.51	146	5.88	0.676
Ophthalmologist	565	16.31	195	19.90	370	14.90	<0.001
Hospital admission	1,108	31.99	344	35.10	764	30.76	0.014
Emergency room	2,713	78.32	772	78.78	1,941	78.14	0.683
People with CVRF at least measured one time							
Systolic blood pressure	2,441	70.47	684	69.80	1,757	70.73	0.586
Diastolic blood pressure	2,441	70.47	684	69.80	1,757	70.73	0.586
Capillary glycemia	922	54.01	246	51.79	676	54.87	0.252
HbA1c	2,923	84.38	775	79.08	2,148	86.47	<0.001
Cholesterol	3,378	97.52	947	96.63	2,431	97.87	0.036
LDL-c	3,310	95.55	922	94.08	2,388	96.14	0.008
HDL-c	3,345	96.56	934	95.31	2,411	97.06	0.011
Triglycerides	3,360	97.00	941	96.02	2,419	97.38	0.034
Waist circumference	266	7.68	57	5.82	209	8.41	0.01
Physical activity (missing values: 2,121)	918	26.50	235	23.98	683	27.50	<0.001
Adequate nutrition (missing values: 2,092)	1,205	34.79	359	36.63	846	34.06	0.162
Treatment adherence (missing values: 2,072)	1,374	39.67	393	40.10	981	39.49	0.031
Electrocardiogram	432	12.47	121	12.35	311	12.52	0.889
Influenza vaccination	1,953	56.38	590	60.20	1,363	54.87	0.004
Risk of diabetic food (Diabetic subjects: 1,707)	306	17.93	90	18.95	216	17.53	0.495
People with at least one drug prescription							
Antihypertensive	125	3.61	32	3.27	93	3.74	0.496
Diuretics	1,372	39.61	531	54.18	841	33.86	<0.001
Beta-Blockers	2,897	83.63	779	79.49	2,118	85.27	<0.001
CCBs	718	20.73	248	25.31	470	18.92	<0.001
ACE-I/ARBs	2,687	77.57	747	76.22	1,940	78.10	0.233
Lipid modifying agents	3,272	94.46	885	90.31	2,387	96.10	<0.001
Antidiabetics	3,241	93.56	913	93.16	2,328	93.72	0.548
Antiplatelet agents	3,325	95.99	913	93.16	2,412	97.10	<0.001
Vitamin K antagonists	286	8.26	98	10.00	188	7.57	0.019
Direct thrombin inhibitors	39	1.13	9	0.92	30	1.21	0.467
Direct factor Xa inhibitors	324	9.35	123	12.55	201	8.09	<0.001
Pharmacological burden (mean, sd)*	10.97	3.78	12.33	3.83	10.44	3.63	<0.001

*N*, number %: percentage; *p*, statistical significance *p* < 0.05. Pearson's Chi-squared test. Student's *T*-test. CVRF, cardiovascular risk factor monitoring; HbA1c, glycated haemoglobin; LDL-c, low-density lipoprotein-cholesterol; HDL-c, high-density lipoprotein-cholesterol; CCB, calcium channel blockers; ACE-I, angiotensin-converting enzyme inhibitors; ARB, angiotensin receptor blocker; ATC, anatomical therapeutic chemical.

* Continuous variables were expressed as mean, standard deviation (sd).

### 3.3 CVRF monitoring

Men had higher rates of monitoring for several CVRFs (Table 2), including glycated hemoglobin (HbA1c), low-density lipoprotein cholesterol (LDL-c), and high-density lipoprotein cholesterol (HDL-c). Total cholesterol, triglycerides and waist circumference were also monitored more frequently among men, with smaller differences. Engagement with physical activity was reported more frequently by men (*p* < 0.001), while women were more likely to receive influenza vaccinations (*p* = 0.004). No significant differences were found in nutritional habits or treatment adherence (Table 2).

### 3.4 Pharmacological treatment

Men were more likely to receive the main guideline-recommended drugs, including beta-blockers, lipid modifying agents, and antiplatelet agents (*p* < 0.001) (Table 2). However, women were more frequently prescribed some concomitant medications, such as diuretics, CCBs, vitamin K antagonists, and direct Factor Xa inhibitors (*p* < 0.001), and showed a higher overall pharmacological burden (*p* < 0.001).

### 3.5 Multivariate analyses

Logistic regression analyses, shown in Table 3, revealed significant gender differences. After age adjustment, logistic regression indicated that most gender differences in health services were no longer statistically significant, with the exception of endocrinologist visits, which remained higher for women (adjusted Odds Ratio: 1.41, 95% Confidence Interval: 1.10–1.80). Significant gender differences remained in CVRFs monitoring: women were less likely to achieve optimal blood pressure levels, HbA1c levels, and waist circumference measurements. They also had lower odds of reporting regular physical activity (adjusted OR: 0.67, 95% CI: 0.52–0.87). Differences in lipid profiles and influenza vaccination were narrowed after adjustment for age. After adjusting for age, women continued to have lower odds of receiving beta-blockers, lipid modifying agents, and antiplatelet agents. In contrast, they were significantly more likely to be prescribed diuretics.

**Table 3:** Crude and age-adjusted odds ratios for health services utilization, risk factors monitoring control and pharmacological treatment in women compared with men.

Variables	Crude		Adjusted	
	Odds Ratios	95% CI	Odds Ratios	95% CI
Primary care	1.58	0.23–30.91	1.05	0.15–21.10
General practitioner	0.59	0.17–2.32	0.58	0.16–2.38
Nurse	1.46	1.10–1.97*	1.13	0.84–1.54
Specialty care	0.60	0.33–1.13	0.66	0.35–1.28
Cardiologist	0.54	0.39–0.74*	0.87	0.63–1.22
Endocrinologist	1.22	0.96–1.54	1.41	1.10–1.80*
Vascular surgeon	0.77	0.55–1.07	0.78	0.55–1.10
Nephrologist	0.93	0.67–1.28	0.75	0.53–1.07
Ophthalmologist	1.42	1.17–1.72*	1.11	0.90–1.35
Hospital admission	1.22	1.04–1.42*	1.00	0.85–1.18
Emergency room	1.04	0.87–1.25	0.89	0.74–1.07
Systolic blood pressure	0.96	0.81–1.12	0.83	0.70–0.98*
Diastolic blood pressure	0.96	0.81–1.12	0.83	0.70–0.98*
Capillary glycemia	0.98	0.84–1.15	0.80	0.67–0.94*
HbA1c	0.59	0.49–0.72*	0.77	0.63–0.94*
Cholesterol	0.63	0.40–0.98*	0.80	0.50–1.27
LDL-c	0.64	0.46–0.90*	0.80	0.57–1.14
HDL-c	0.61	0.42–0.90*	0.83	0.56–1.23
Triglycerides	0.65	0.44–0.98*	0.87	0.57–1.33
Waist circumference	0.67	0.49–0.90*	0.70	0.51–0.96*
Physical activity	0.62	0.49–0.80*	0.67	0.52–0.87*
Adequate nutrition	1.30	0.91–1.91	1.08	0.74–1.61
Treatment adherence	6.81	1.39–122.92*	4.84	0.96–88.24
Electrocardiogram	0.98	0.78–1.23	0.99	0.78–1.25
Influenza vaccination	1.24	1.07–1.45*	0.87	0.74–1.03
Risk of diabetic food	1.06	0.82–1.37	0.87	0.66–1.14
Antihypertensive	0.87	0.57–1.29	0.75	0.48–1.13
Diuretics	2.31	1.99–2.69*	1.52	1.29–1.80*
Beta-Blockers	0.67	0.55–0.81*	0.75	0.62–0.92*
CCBs	1.45	1.22–1.73*	1.19	0.99–1.43
ACE-I/ARBs	0.90	0.76–1.07	0.83	0.70–1.00
Lipid modifying agents	0.38	0.28–0.51*	0.57	0.42–0.78*
Antidiabetics	0.91	0.68–1.23	0.96	0.71–1.31
Antiplatelet agents	0.41	0.29–0.57*	0.48	0.33–0.68*
Vitamin K antagonists	1.36	1.05–1.75*	1.09	0.83–1.42
Direct thrombin inhibitors	0.76	0.34–1.54	0.64	0.28–1.34
Direct factor Xa inhibitors	1.63	1.28–2.06*	1.00	0.77–1.29

95% CI: Confidence interval 95%. HbA1c, glycated haemoglobin; LDL-c, low-density lipoprotein-cholesterol; HDL-c, high-density lipoprotein-cholesterol; CCB, calcium channel blockers, ACE-I, angiotensin-converting enzyme inhibitors; ARB, angiotensin receptor blocker.

* Statistical significance *p* < 0.05.

### 3.6 Oaxaca–Blinder decomposition analysis

The Oaxaca-Blinder decomposition analyses assessed the contribution of observed variables to gender inequalities. So, variables with negative values reduced the observed inequalities, while those with positive values increased them (Figuress 2, 3). Complete data from the OAXACA decomposition analyses, such as the explained fraction of the models and the contribution of each variable, can be found in the Supplementary Tables S1 and S2.

**Figure 2:**
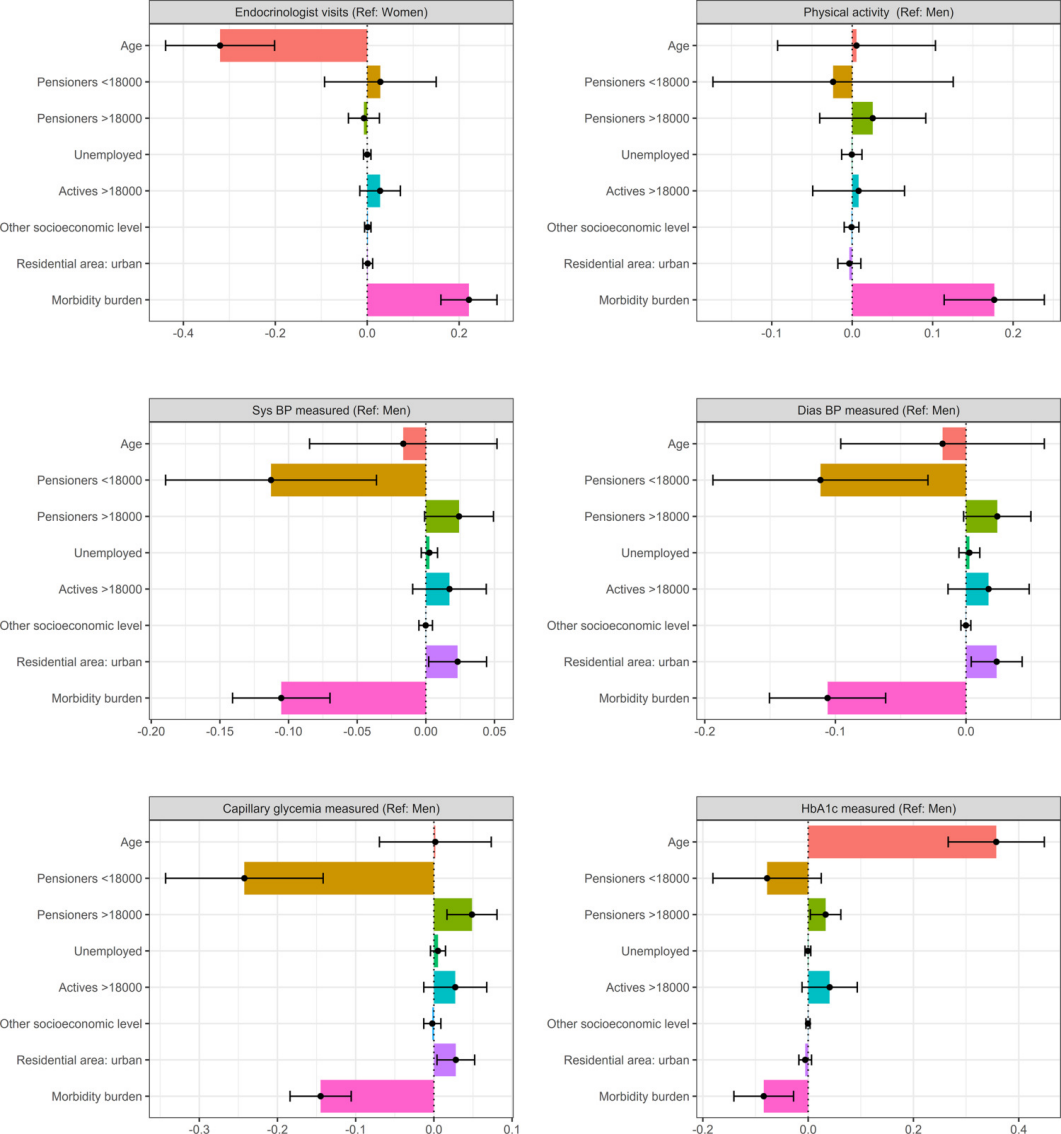
Decomposition of gender inequalities in healthcare utilization and CVRF monitoring. Oaxaca decomposition analyses. CVRF, cardiovascular risk factor; Ref, reference (gender taken as reference category); Sys BP, systolic blood pressure; Dias BP, diastolic blood pressure; HbA1c, glycated haemoglobin; categories of reference: actives <18,000, rural residence.

**Figure 3:**
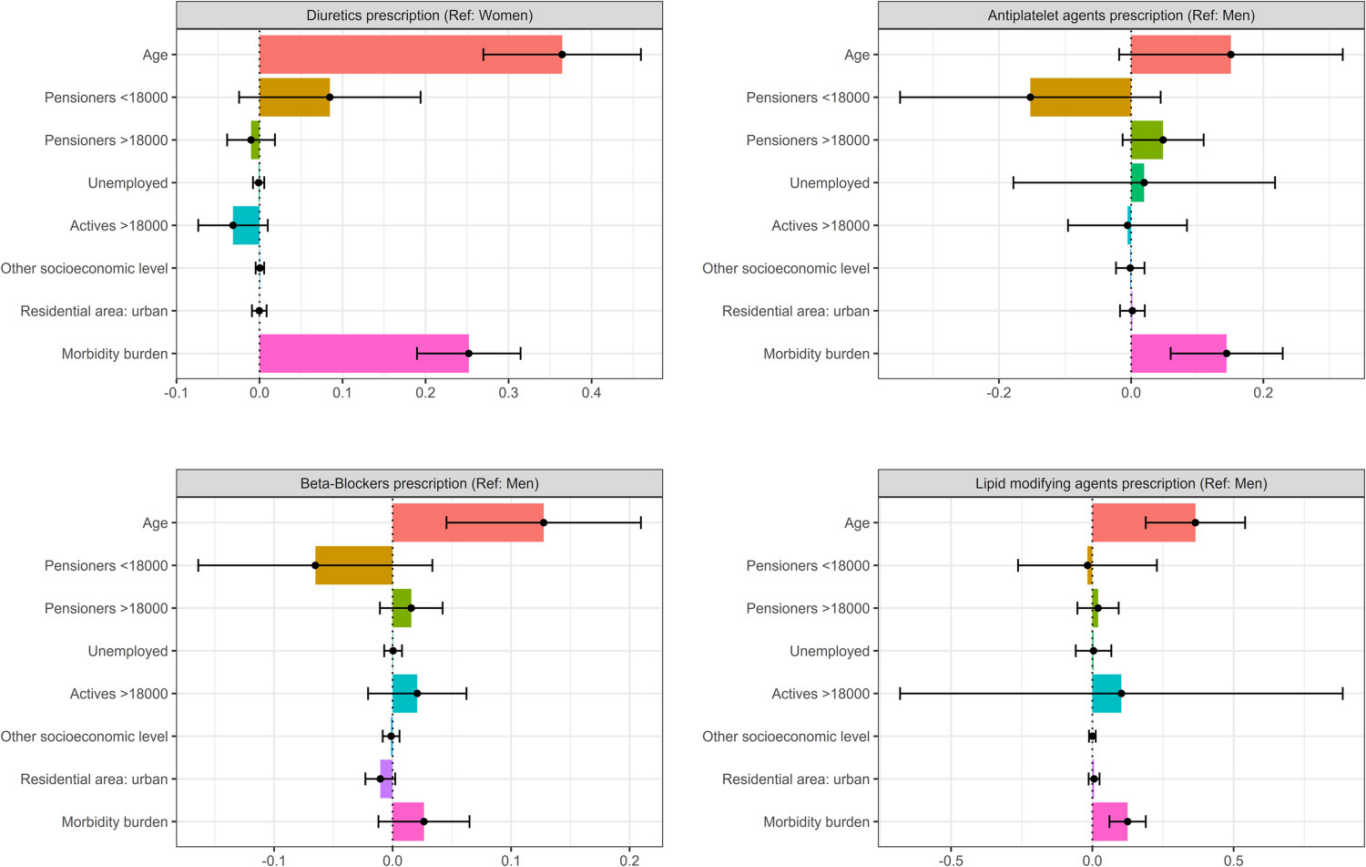
Decomposition of gender inequalities in treatment prescribing patterns. Oaxaca decomposition analyses. CVRF, cardiovascular risk factor; Ref, reference (gender taken as reference category); Sys BP, systolic blood pressure; Dias BP, diastolic blood pressure; HbA1c, glycated haemoglobin; categories of reference: actives <18000, rural residence.

Regarding healthcare utilization, the OAXACA analysis revealed that only 19.74% of the observed gender inequalities in endocrinology visits were explained by the variables included in the model, with age and morbidity burden being the main contributing factors. The lower age of men mitigated these inequalities, while the lower morbidity burden of men with respect to women increased the existing differences.

Concerning physical activity, which was more reported in men, the lower morbidity burden of men was the main explanatory factor of these gender differences, along with their higher socioeconomic status. However, being a low-income pensioner, seemed to mitigate these differences, while age did not seem to influence gender differences.

In terms of CVRF monitoring, the explained fraction varied from 63.15% for HbA1c to 38.78% for physical activity. The explanatory variables in the model exhibited different effects on each of the CVRF studied. With regard to blood pressure, which was less monitored in women, the factors that contributed most to increasing gender inequalities were urban residence and high socioeconomic status, considering actives <18,000€ as reference. In contrast, the older age of women, being a low-income pensioner, and their higher morbidity burden in this study reduced these differences. A similar explanatory pattern was observed for capillary glycemia measurement, except that age did not appear to be associated with variations in gender differences observed. For HbA1c measurement, the factors more associated with increased gender inequalities were age, as the main contributing factor, and high socioeconomic status. In contrast, being a low-income pensioner and morbidity burden appeared to reduce these inequalities.

Regarding drug prescription, the explained fraction of observed gender differences varied across the pharmacological groups analyzed. The explanatory fraction of the model was high in diuretics and lipid modifying agents (more than 50%) while the explanatory factor of beta-blockers and antiplatelet agents was low, suggesting the influence of additional factors not considered in the model. As shown in Figuress 2, 3, age and morbidity burden were the main factors contributing to gender inequalities in the prescribing of guideline-recommended drugs. Regarding age, the older age of women was related with increasing gender differences in the prescription of antiplatelets, beta-blockers and lipid modifying agents. On the contrary, the younger age of men seemed to increase prescription differences in diuretics. Regarding socioeconomic status, none of the categories analysed showed a statistically significant effect on drug prescription, but it was possible to observe some associations. In the case of antiplatelets and beta-blockers, the high frequency of women that were pensioners with low income reduced the inequalities observed. In the case of diuretics, with a higher prescription in women, the lower frequency of men with low income increased the differences. Finally, the morbidity burden increased the observed differences in all the drugs considered. The lower morbidity burden of men increased the differences observed in men and women in the prescription of diuretics. On the other hand, the higher morbidity burden of women increased the differences in the prescription of antiplatelets, beta-blockers and lipid-modifying agents.

## 4 Discussion

### 4.1 Sociodemographic and clinical profile differences

In our population following a first AMI, men and women had different socioeconomic and clinical characteristics. Women were older, more frequently institutionalized, and had higher morbidity burden and lower socioeconomic status. These factors are essential in order to comprehend the observed inequalities in healthcare utilization, CVRF monitoring, and treatment.

### 4.2 Healthcare service utilization

Nearly all patients had at least one primary care visit within the first year after AMI, as expected, since primary care is the principal setting for health care follow-up (23). However, women visited primary care nurses more frequently than men, as described in the literature (24–26) and may be due to differences in health-seeking behaviors or greater awareness of prevention (24). In this sense, a study by Vallejo-Torres et al. found that nurse utilization was associated with older age, female gender, the presence of more chronic conditions, and lower socioeconomic status (26) which is in line with our results.

In terms of specialist care, women were more likely to visit endocrinologists but had lower overall rates of visits to other specialists, particularly cardiologists, as the evidence shows (24,27). Our prior scoping review (28) suggested that this lower referral rate to cardiology may be related to an androgenic bias in cardiovascular care and under-recognition of the disease in women by both patients and healthcare providers (27,28), as well as, lower awareness of CVD, less social support, or lower socioeconomic status (29).

After adjusting for age, only endocrinologists' visits remained significant. Oaxaca decomposition attributed this to women's higher morbidity burden, which increased the observed differences.

This could be partly explained by the higher prevalence of endocrine disorders in women, particularly postmenopausal and elderly, such as thyroid disease, osteoporosis, or hormonal imbalances (30–32), which. often coexist with other comorbidities that increase cardiovascular risk and complicate disease management (33–36). In contrast, men's younger age helped reduce the differences observed. However, the association between age and referral patterns in older women is complex, and may be influenced by unmeasured factors such as race or marital status (37).

Hospital admissions were more frequent among women, however, became not significant after adjusting for age. These findings are consistent with previous studies (38,39), suggesting that while unadjusted data may show higher admission rates among women, factors such as age and other social determinants play a significant role in the observed differences.

### 4.3 CVRF monitoring

Gender inequalities in CVRF monitoring were pronounced. Men had a higher frequency of recorded risk factor monitoring, including blood pressure, glycemic control (HbA1c), and cholesterol levels (8, 40–42). These findings align with existing literature suggesting a potential gender bias in management, with women receiving less frequent CVRF monitoring and more conservative treatments, despite being older and having a higher morbidity burden (43). Urban residence increased gender inequalities in CVRF management, which could be explained by differences in access to health care. Rural areas often have shorter waiting lists and greater availability of primary care services (44–47). The relationship between socioeconomic status and gender inequalities in health outcomes is somewhat controversial. We observed a reduction in gender inequalities in blood pressure, capillary glycemia, and HbA1C control associated with lower socioeconomic status in women. This could be related to the fact that women with lower socioeconomic status in our population were older and had a higher burden of disease, leading them to visit primary care more frequently. As a result, their CVRFs were monitored more regularly, narrowing the gap between men and women. Some authors support this explanation (48,49). Conversely, other studies suggest that lower socioeconomic status may limit women's CVRFs monitoring, thereby increasing gender inequalities (50,51). Also, other studies have associated higher socioeconomic status with better CVRF monitoring and greater access to health care, highlighting the complexity of this relationship (29,52,53).

For HbA1c measurement, age has an important role in increasing existing inequalities, which has been related with the older age of women (54–57). In terms of lifestyle practices, women reported greater adherence to healthy lifestyle recommendations, such as proper diet and immunizations. This is consistent with existing literature (58–63). For physical activity, consistent with what has been reported in the literature (60,61,64,65), women in our study were less likely to report practicing physical activity than men. Oaxaca analysis identified that morbidity burden and high socioeconomic status increased gender inequalities in physical activity practice, whereas lower income reduced inequalities. As has been widely described, gender, age and socioeconomic status are known to be important determinants of physical activity (66). While higher income has been associated with greater physical activity (67), in low-income settings, men and women have shown to face similar barriers to physical activity, reducing gender inequalities (68). In addition, chronic health conditions negatively impact physical activity levels, with women experiencing greater reductions, further widening inequalities (69).

### 4.4 Pharmacological treatment

Women were less likely to be prescribed guideline-recommended medications and were more likely to receive adjunctive therapies, as diuretics. These results are consistent with the literature (8,28,41,42,70,71). The Oaxaca analysis showed that older age and higher morbidity burden were the main factors contributing to this gender inequalities. This is in line with some studies that have found strong associations between age, morbidity burden and greater risk of exacerbations, leading to under-prescription of the pharmacological groups studied, increasing gender inequalities (24,48,72–74). Similarly, the large international PRAISE registry (75) reported lower prescription rates of guideline-recommended therapies in women following acute coronary syndromes. However, it also found that female gender was not associated with an increased risk of adverse outcomes, including bleeding, highlighting the need for more intensive, evidence-based treatment strategies in women based on their clinical profile rather than gender alone. The analysis of socioeconomic status reveals complex interactions with gender disparities in the prescribing patterns of guideline-recommended medications, as explored in our previous research (70). While higher socioeconomic status is generally associated with reduced gender disparities (29,52,76), our study found that lower income subjects, particularly older women with a higher morbidity burden, experienced a narrowing of these disparities in the prescription of antiplatelets and beta-blockers, although this association was not statistically significant. This suggests that women in our population may receive more clinical attention, leading to more equitable prescribing patterns of guideline-recommended therapies (48,50,77).

Regarding diuretic prescription, our findings align with existing research associating lower socioeconomic status with higher diuretic prescription rates among women, which could be associated with the management of multiple comorbidities (78). Studies indicate that women with low socioeconomic status are more susceptible to polypharmacy, with diuretics frequently prescribed as part of a prescribing cascade (79). Additionally, diuretic use tends to increase with the overall number of medications prescribed, which corresponds to the greater morbidity burden observed in our study population (80).

### 4.5 Strengths and limitations

One of the major strengths of this study is the use of the CARhES cohort, a population cohort of RWD from the Aragon Health Service. This increases the internal validity of the study by ensuring a representative study population at the regional level.

In addition, a key strength of our study is the use of the Oaxaca-Blinder decomposition method to examine gender inequalities. This analytical approach allows us to separate observed gender differences into two components: an explained fraction, attributable to measurable variables, and an unexplained fraction, which may reflect differential effects of these characteristics, structural or behavioural inequalities, or potential discrimination (20). When we apply this method from a gender perspective, our analysis provides a more nuanced and comprehensive understanding of the mechanisms underlying inequalities in the use of services and management of CVD.

Nonetheless, certain limitations need to be acknowledged. A limitation of using registered diagnoses is the potential for diagnostic bias in CVD, as it excludes undiagnosed subjects. As highlighted in the literature (43,72), this issue could be particularly relevant for women because of potential underdiagnosis related to the nonrecognition of their symptoms in previous clinical guidelines. Another potential limitation of the study is the exclusion of patients who died during the index event, which may introduce survival bias. While the mortality rates were comparable between sexes, women represented a slightly higher proportion of the deceased and were, on average, older. No significant sex differences were observed in terms of morbidity burden or number of chronic conditions. However, the exclusion of these cases may have led to an underrepresentation of more severe female cases. Furthermore, the established follow-up period of one year may have further contributed to survival bias, as it excludes patients with shorter survival, potentially affecting older and more vulnerable subjects, particularly women. This decision was made to ensure a consistent observation period for all participants, thus minimising variability in follow-up duration and enabling reliable analysis of post-AMI care. Another limitation is the lack of information regarding the actual reason for the patient's visit. As a result, we cannot confirm whether consultations were directly related to the AMI episode. Finally, data on key lifestyle parameters, such as adequate diet, physical activity, smoking, or adherence to treatment, are underreported in electronic health records, as we have shown in our results. Nevertheless, we have chosen to present this information to highlight both their potential impact and the significant gaps in information registration.

## 5 Conclusions

Our study showed the existence of gender inequalities in post-AMI care, particularly in CVRFs monitoring and pharmacological treatment., Women visited more frequently primary care nurses and endocrinologists, while men visited more cardiologists. CVRFs were less frequently monitored in women, although they had better adherence to preventive and lifestyle behaviors. In addition, the main guidelines recommended drugs were prescribed to women less frequently. These inequalities were mainly explained by age, morbidity burden and socioeconomic status. Specifically, older age and higher comorbidity burden among women resulted in lower prescription of recommended drugs. Conversely, older age, lower income, and higher morbidity burden appeared to reduce differences in CVRF monitoring. However, a significant part of the observed differences remained unexplained, suggesting the presence of underlying systemic or structural biases in care delivery.

### 5.1 Research implications

It is important to assess whether factors such as age or comorbidities justify the reduced care observed or, on the contrary, reflect bias in clinical decisions.

Raising awareness of gender differences is essential to ensure equitable care. For example, better recording of CVRFs and lifestyle habits in health records is key to improving personalized care. Clinical practice guidelines must incorporate a gender-sensitive approach to ensure equitable and personalized care, as current recommendations do not fully address the differentiated healthcare needs of women, contributing to treatment inequities. Beyond clinical practice, these findings point to wider systemic challenges, including potential barriers that women face in accessing cardiovascular care as explored in our previous scoping review (28) and by Giordano et al. (11). Several social and cultural factors—like unrecognized symptoms, caregiving duties, fear of being a burden, low income, limited education, and reduced autonomy—can delay or prevent women with AMI from getting timely care. The establishment of follow-up protocols or structured monitoring programs, especially in primary care, can reduce the gender gap and improve health outcomes.

Finally, gender analysis is complex and closely linked to other social factors, making it hard to explain all the causes of disparities. This highlights the need for more research using an intersectional approach.

## Data Availability

The original contributions presented in the study are included in the article/Supplementary Material, further inquiries can be directed to the corresponding author/s.
