# Supplementary material for: When gender matters: inequalities in health services utilization and risk factors monitoring after acute myocardial infarction
Source: Front Glob Womens Health. 2025 Jun 26;6:1605400. doi: 10.3389/fgwh.2025.1605400 (PMC12241081; doi:10.3389/fgwh.2025.1605400)
Supplement: Supplementary file 1 [file Table1.docx]

**Supplementary material Table s1. Oaxaca decomposition fractions.**

|  | **Explained fraction (%)** | **Unexplained fraction (%)** |
| --- | --- | --- |
| **Endocrinologist visits** | 19.74 | 80.26 |
| **Exercise** | 38.78 | 61.22 |
| **Systolic and Dyastolic Blood pressure** | 42.02 | 57.98 |
| **Capilary glycaemia** | 49.02 | 50.98 |
| **HbA1c** | 63.15 | 36.85 |
| **Diuretics prescription** | 67.81 | 32.18 |
| **Antiplatelet agents’ prescription** | 12.10 | 87.89 |
| **Beta-blockers prescription** | 27.66 | 72.34 |
| **Lipid modifying agents’ prescription** | 98.85 | 1.15 |
