# Supplementary material for: When gender matters: inequalities in health services utilization and risk factors monitoring after acute myocardial infarction
Source: Front Glob Womens Health. 2025 Jun 26;6:1605400. doi: 10.3389/fgwh.2025.1605400 (PMC12241081; doi:10.3389/fgwh.2025.1605400)
Supplement: Supplementary file 2 [file Table2.docx]

**Supplementary material Table s2. Oaxaca decomposition variable contribution.**

| **Variable contribution (%)** | **Endocrinologist visits** | **Exercise** | **Systolic and Diastolic Blood Pressure** | **Capilary glycaemia** | **HbA1c** | **Diuretics prescription** | **Antiplatelet agents’ prescription** | **Beta-blockers prescription** | **Lipid modifying agents’ prescription** |
| --- | --- | --- | --- | --- | --- | --- | --- | --- | --- |
| Age | 52.68 | 0.40 | 5.29 | 0.40 | 59.50 | 48.61 | 28.94 | 47.69 | 59.90 |
| Actives >18,000€ | 4.52 | 3.39 | 5.71 | 5.47 | 6.79 | 4.29 | 1.02 | 7.66 | 16.02 |
| Unemployed | 0.00 | 0.23 | 0.85 | 1.05 | 0.09 | 0.15 | 3.73 | 0.15 | 0.65 |
| Pensioners < 18,000€ | 5.24 | 8.88 | 37.50 | 48.54 | 13.09 | 11.58 | 29.09 | 24.56 | 2.75 |
| Pensioners > 18,000€ | 1.54 | 9.51 | 8.12 | 9.65 | 5.62 | 1.52 | 9.42 | 6.11 | 3.25 |
| Other socioeconomic level | 0.11 | 0.08 | 0.02 | 0.28 | 0.03 | 0.02 | 0.15 | 0.26 | 0.02 |
| Urban residence | 0.17 | 1.26 | 7.67 | 5.62 | 0.92 | 0.04 | 0.37 | 3.79 | 0.96 |
| Morbidity burden | 35.72 | 75.95 | 34.84 | 28.99 | 13.96 | 33.79 | 27.29 | 9.79 | 19.45 |
